# Supplementary material for: Utilization of family planning and associated factors among women with disabilities in ethiopia: A systematic review and meta-analysis
Source: PLoS One. 2023 Sep 8;18(9):e0291189. doi: 10.1371/journal.pone.0291189 (PMC10490906; doi:10.1371/journal.pone.0291189)
Supplement: S2 File — (PDF) [file pone.0291189.s003.pdf]

Additional file 2: Newcastle-Ottawa Quality Assessment Scale to assess the utilization of family planning among women with disabilities in Ethiopia

**Studies**	**Selection**				**Comparability**	**Outcome**		**Total**
	Representativeness- (1)	Sample size- (1)	Non-respondents (1)	Ascertainment of the exposure (risk factor)- (2)	The subjects in different outcome groups are comparable - (2)	Assessment of the outcome - (2)	Statistical test- (1)	
Yesgat YM, et al.	*	*	*	*	**	**	*	9
Mekonnen AG, et al.	*	*	*	*	**	**	*	9
Beyene GA, et al.	*	*	–	*	**	**	*	8
Yimer AS, et al.	*	*	*	*	*	**	*	8
Abera S (upulished)	*	*	*	**	**	*	*	9
Tilahun A (upulished)	*	*	*	*	**	**	*	9
Tsegay K, et al.	*	*	*	**	*	*	*	8

Interpretation of the score: Very Good Studies: 9-10 points, Good Studies: 7-8 points, Satisfactory Studies: 5-6 points and Unsatisfactory Studies: 0 to 4 points
