# Supplementary material for: Utilization of family planning and associated factors among women with disabilities in ethiopia: A systematic review and meta-analysis
Source: PLoS One. 2023 Sep 8;18(9):e0291189. doi: 10.1371/journal.pone.0291189 (PMC10490906; doi:10.1371/journal.pone.0291189)
Supplement: Attachment — Submitted filename: Response to Reviewers.docx [file pone.0291189.s004.docx]

The authors would like to thank the editorial team and team of reviewers for constructive and valuable comments. The authors are very happy to submit the revised version of the manuscript entitled “Utilization of Family Planning and Associated Factors among Women with Disabilities in Ethiopia: a systematic review and meta-analysis” for its publication in your Journal. The comments of the editors and the reviewers were highly insightful and enabled us to greatly improve the quality of our manuscript. In this revised manuscript we made substantial changes to address your concerns in a point-by-point response. We are very keen to incorporate further comments, if any, for the betterment of the final manuscript.

**Response to the Editor**

**Methods**

1. You said the PROSPERO registration is on-going, what is the importance of registering it after you have published the review?

**Answer** –Thank you for your comment; sorry for the inconvenience, and it is corrected in the revised manuscript.

2. It is better of specify the number of articles you have gotten from each data bases (Pub Med/MEDLINE=? Google Scholar=? African Journal of Online (AJOL)=? CINAHL=?, HINARI=?, Scopus=?,Science Direct, Excerpta Medica database (EMBA, SE), DOAJ, Web of Science, Google, and other organization's websites=?)

**Answer** – Thank you for your question. Here are the number of articles from each data base Pub Med/MEDLINE=681 Google Scholar=426 African Journal of Online (AJOL) =36 CINAHL=211, HINARI=191, Scopus=86, Science Direct=62, Excerpta Medica database (EMBA, SE) = 113, DOAJ= 38, Web of Science=26, Google= 271 and organization's websites=2)

3. What is the final mesh term used in each searching engines?

**Answer –**Thank you for your questions; you can see additional file 1 to understand the whole search engine by one of the most usable data base PubMed.

Here are some of the mesh terms that are used in searching engines.

| Word | Mesh terms |
| --- | --- |
| Utilization of Family Planning | Family planning utilization, use of family planning methods, utilization of family planning services, State of family planning, Family Planning Service Utilization, practice of family planning, Providing family planning services, Contraceptive utilization |
| Factors associated with family planning | Influencing Factors, associated factors, factors, factors influencing, |
| Women with disabilities | Disabled women, disables , Women with disability, disability, disabilities, |

4. Is publication year or study period better indicating the time period of the articles?

**Answer –**Thank you for your questions; the study period is better indicating the time period of the articles and publication year also used to determine how old the information can be.

**Result**

1. What does it mean p = 0.000?

**Answer –**Thank you for your comment; it is an editorial problem and corrected in the revised manuscript

2. You have checked three independent variables to assess their association with the outcome variable, some single articles revealed more than three significant variable.

**Answer –** Thank you for your inquiry; you are right that some single articles revealed more than three significant variable. But the only factor identified as a significant factor in the two and above primary studies was included in this review and meta-analysis. That is the reason for only three independent variables were assessed for their association.

3. The discussion is shallow, it needs to entertain more perspectives

**Answer –**Thank you for your comment; it is accepted and corrected in the revised manuscript.

**Response to the reviewer**

**Reviewer #1:** the Meta analysis is very important as a disability is a topic very poorly reported in current literature. This study underlines in Ethiopia those aspects in women with disabilities encountering pregnancy.

**Answer –**Thank you for your insightful idea.

**Reviewer #2:**

A. Acknowledge for addressing disadvantaged segment of the community.

**Answer –**Thank you for your idea.

B. Scope: The scope of the issue and the outcome variables needs re-operationalization. The authors failed to include fertility.

**Answer –** Thank you for your comment; it is accepted and corrected on page 5 line 22-28 of the revised manuscript.

C. Methods: Whether retracted articles are used in the analysis were not mentioned,

**Answer –** Thank you for your comment; as mentioned in the Prisma diagram(figure 1), 13 articles were retracted to assess their eligibility, but only seven studies were included in the analysis and the other six articles were excluded because of the outcome of interest is not reported. It is also described on page 6, line 19-24 of the revised manuscript.

D. Illegibility: Not included all observational studies.

**Answer –** Thank you for your inquiry; First the authors plan to include all observational studies but all studies who fulfil the eligibility criteria in this review were crossectional, that’s why the authors don’t included all observational studies.

E. Whether all studies defined disability in the similar way: Not explained

**Answer –** Thank you for your suggestion, majority of the studies included in this review defined people with disability as women having hearing, visual and physical impairments or limb defects (1–5). This is described in the revised manuscript on page 5, line 23-24.

F. Data analysis and Presentation: Inadequate.

**Answer –** Thank you for your comment. The authors carried out all of the necessary analysis to show the findings of the meta-analysis, such as a random effects model that was used to determine the pooled prevalence of family planning utilisation, as shown in **Figure 2.** Because there is heterogeneity across studies, meta-regression and subgroup analysis were used to identify the source of heterogeneity using the sample size, year of study, and region, as shown in Figures **4A and 4B.** A funnel plot and Egger's test were used to check for publication bias, as shown in Figure 3 and **Table 2.** A leave-one-out sensitivity analysis was also performed to see how individual studies affected the pooled estimate of family planning utilisation. The results showed that removing a single study had no significant effect on pooled prevalence, as shown in **Table 3.**

G. Authorship: inconsistent in the main document and in the methods section.

**Answer –** Thank you for your comment; it is accepted and corrected in the revised manuscript.

H. Language and statistic: Need major revision.

**Answer –** Thank you for your feedback; it has been accepted, and the paper has been revised by language experts and online writing tools like Grammarly and QuillBot. As previously stated, this study performed all of the necessary analysis in order to show the results of the meta-analysis.

Reference

1.	Beyene GA, Munea AM, Fekadu GA. Modern contraceptive use and associated factors among women with disabilities in gondar city, amhara region, north west ethiopia: A cross sectional study. Afr J Reprod Health. 2019;23(2):101–9.

2.	Kellali T, Hadush G FH. Modern Contraceptive Methods Utilization and Associated Factors among Women with Disabilities. Int J Pharm Biol Sci Fundam [Internet]. 2017;13(01)(01):1–8. Available from: www.ijpbsf.com

3.	Mekonnen AG, Bayleyegn AD, Aynalem YA, Adane TD, Muluneh MA, Asefa M. Level of knowledge, attitude, and practice of family planning and associated factors among disabled persons, north-shewa zone, Amhara regional state, Ethiopia. Contracept Reprod Med. 2020;5(1):1–7.

4.	Mesfin Yesgat Y, Gebremeskel F, Estifanous W, Gizachew Y, Jemal S, Atnafu N, et al. <p>Utilization of Family Planning Methods and Associated Factors Among Reproductive-Age Women with Disability in Arba Minch Town, Southern Ethiopia</p>. Open Access J Contracept. 2020;Volume 11:25–32.

5.	Abera S. The Assessment of Determinants of family planning use and unmet need among women of reproductive age group with disabilities in Addis Ababa. 2016;(November).
