# Supplementary material for: Utilization of family planning and associated factors among women with disabilities in ethiopia: A systematic review and meta-analysis
Source: PLoS One. 2023 Sep 8;18(9):e0291189. doi: 10.1371/journal.pone.0291189 (PMC10490906; doi:10.1371/journal.pone.0291189)
Supplement: Attachment — Submitted filename: Manuscript-C.docx [file pone.0291189.s005.docx]

**Utilization of Family Planning and Associated Factors among Women with Disabilities in Ethiopia: a systematic review and meta-analysis**

**Authors**

Tesfanesh Lemma^1^*	*Email: tesfitimnt@gmail.com*

Mulualem Silesh^1^ *Email:* [*sileshmulualem22@gmail.com*](mailto:sileshmulualem22@gmail.com)

Birhan Tsegaw Taye^1^ *Email:* [*tsegawbirhan2@gmail.com*](mailto:tsegawbirhan2@gmail.com)

Tebabere Moltot^1^	*Email: mtebabere@gmail.com*

Moges Sisay Chekol ^1^	*Email: sisaymoges07@gmail.com*

Maritu Ayalew^2^	*Email: marayalew4@gmail.com*

**Affiliation**

1. Department of Midwifery, Asrat Woldeyes Health Science Campus, Debre Berhan University, Debre Berhan, Ethiopia.
2. Department of Midwifery, Teda Health Science College, Gondar, Ethiopia.

*Corresponding author:

Tesfanesh Lemma

Email:tesfitimnt@gmail.com. , Telephone No: +251910901201

**Abstract**

**Background:** Persons with disabilities have a right to make their own choices about their bodies, health, and lives, especially regarding their sexual and reproductive health. But they may experience more challenges than women without disabilities in having their reproductive health needs met. So there is an urgent need to scale up disability inclusion in all levels of the health system including family planning. Therefore, the objective of this study was to estimate the pooled prevalence of Family Planning Utilization and Associated Factors among Women with Disabilities in Ethiopia.

**Methodology:** Studies were gathered from Pub Med/MEDLINE, Google Scholar, African Journal of Online (AJOL), CINAHL, HINARI, Scopus, Science Direct, Excerpta Medica database (EMBA, SE), DOAJ, Web of Science, Google, and other organization's websites using a combination of search terms and Boolean operators. The modified Newcastle Ottawa Scale (NOS) for cross-sectional research was used by three authors to independently assess the quality of each study. For statistical analysis, STATATM Version 11 software was employed. For the meta-analysis, the random-effects (Der Simonian and Laird) technique was applied. The heterogeneity test was performed using I-squared (I^2^) statistics. A one-out sensitivity analysis was performed.

**Result:** A total of 7 articles with 2787 participants were included in this systematic review and meta-analysis. The pooled prevalence of family planning utilization among Women with Disabilities was 29.6% (95% CI: 22.3, 36.8); I2 = 94.6%). Women who were in marital union (p<0.001) and who had a discussion with their husbands (p=0.007) were factors significantly associated with the utilization of family planning among women with disabilities.

**Conclusion:** The finding of this study showed that utilization of family planning among women with disability is relatively low. It is critical to pay more attention to the reproductive health care needs of women with disabilities to improve health care equity and quality.

**Keywords:** Utilization, Family Planning, Women with Disabilities, Ethiopia

1. **INTRODUCTION**

A disability is defined as a condition or function judged to be significantly impaired relative to the usual standard of an individual or group. The term is used to refer to individual functioning, including physical impairment, sensory impairment, cognitive impairment, intellectual impairment mental illness, and various types of chronic disease (1). According to the 2021 World Health Organization (WHO) report, Over 1 billion people in the world live with some form of disability (2). Women account for the majority of the disabled population in the world; globally, one in five women lives with a disability compared to one in eight men. In low and middle-income countries, women are estimated to comprise up to three-quarters of persons with disabilities (3).

Family planning is "the ability of individuals and couples to anticipate and attain their desired number of children and the spacing and timing of their births (4). It is the information, means, and methods that allow individuals to decide if and when to have children (5). Access to safe, voluntary family planning is a human right and is critical in ensuring gender equality and women's empowerment, and it is a key factor in reducing poverty and improving livelihoods (5–7). Persons with disabilities have a right to make their own choices about their bodies, health, and lives, especially regarding their sexual and reproductive health and rights and freedom from discrimination and violence (8). They have the same sexual and reproductive health needs and rights as people without disabilities, but often they are not given information about reproductive and sexual health or adequate care (9,10)

Studies indicate that disabled women were at higher risk of experiencing sexual violence than nondisabled women (11–13), and experience more challenges than women without disabilities in having their reproductive health needs met. Women with Disabilities (WWDs) face multiple barriers to quality contraceptive care (14), whether through stigma, increased risk of violence or abuse, lack of access to care (15), prejudices, and discrimination from healthcare service providers (14,16) and receive poor quality services (2,17).

So there is an urgent need to scale up disability inclusion in all levels of the health system, particularly primary health care (2) like family planning(15). According to a review conducted in Low-and Middle-Income countries, contraception use among WWDs ranged from 13 % to 31.1 %, with 24.3 percent of unmet needs (18).

**Objectives of the review**

- To determine the prevalence of family planning utilization among WWDs in Ethiopia.
- To identify the factors associated with family planning utilization among WWDs in Ethiopia.

2. **Methods and materials**
   2.1. **Study design and search strategy**

The purpose of this systematic review and meta-analysis was to assess the pooled prevalence of family planning utilization and associated factors among women with disabilities in Ethiopia using the Preferred Reporting Items for Systematic Reviews and Meta-analyses (PRISMA) statement guidelines (19,20). We searched the PROSPERO database to see whether there were any comparable on-going systematic reviews and meta-analyses.

Three authors searched the following databases for relevant studies: Pub Med/MEDLINE, Google Scholar, African Journal of Online (AJOL), CINAHL, HINARI, Scopus, Science Direct, Excerpta Medica database (EMBA, SE), DOAJ, Web of Science, Google, and other organization's websites by using the full title (Utilization of family planning and associated factors among women with disability in Ethiopia) and using the following searching keywords or terms; ''Family planning utilization'', ''use of family planning methods, ''utilization of family planning services'', ''State of family planning'', ''Family Planning Service Utilization'', ''practice of family planning'', ''Providing family planning services'', ''Contraceptive utilization'', ''Influencing Factors'', ''associated factors'', '' factors influencing'', ''Disabled women, ''disables'', ''Women with disability'', ''disability'', ''disabilities'' and, ''Ethiopia" In order to find any further missed studies, the reference lists of all included published and unpublished studies were reviewed. All fields and MeSH (Medical Subject Headings) terms with Boolean operators ("OR" and/or "AND") were used to search studies in the advanced PubMed search engine (**Additional file 1**).

2.2. **Eligibility criteria**

Both published and unpublished observational studies in English that report the prevalence and/or associated factors of family planning utilization among disabled women in Ethiopia were included. This review also included studies done up until November 15, 2022. Studies with a different operational definition or outcome of interest, as well as those whose full texts were unavailable, were excluded.

2.3. **Outcome measurement, study selection, and quality assessment**

The primary outcome of this review was the pooled prevalence of family planning utilization. The second outcome of this review was the associated factors of family planning utilization. The only factor identified as a significant factor in the two and above studies was included in this review and meta-analysis. After studies were searched from different international databases and organization websites, studies were screened by using the following criteria (duplication, relevancy, accessibility of full text, and outcomes of interest). Finally, the quality of each study was assessed using the standard quality assessment tool [Newcastle-Ottawa Scale (NOS)] (21) and was assessed by five authors (TL, BTT, TM, MSC, and MA) independently using the following components: selection, comparability, and outcome; which were graded by five stars, two stars, and three stars respectively. Any disagreements between the five authors during quality appraisal were resolved by another author (MS) through discussion and re-evaluation of selected studies. For analysis, only the primary studies with a medium score (fulfilling 50% of the quality evaluation criteria) and above were included (22) (**Additional file 2**).

**Operational definition**

**Women with disability: -** women having hearing, visual and physical impairments or limb defects(23)(24).

**Family planning utilization: -** The women with disability had ever used any modern method of contraceptive during her sexual life and or she is current user (23–25).

**Total fertility rate (TFR):-** is the measure of children a women would have over her life time if she were to follow current age-specific fertility rates (25).

**Data extraction process**

Three independent authors (TL, MS, and BTT) extracted the data using a data extraction format prepared in a Microsoft Excel 2010 spreadsheet. The following information was extracted: the first author's name, the year of study, the study area, the Region, the study design, the sample size, the sampling method, the prevalence of family planning, and the associated factors with their odds ratios. Differences during data extraction were resolved through discussion and consensus by involving the fourth author (TM).

2.4. **Data synthesis and statistical analysis**

For analysis, STATA Version 11 software was used. Because high heterogeneity across studies was identified using inverse variance (*I*^2^) statistics with its corresponding p-value (*I*^2^ = 94.6%, *p* < 0.001) (26–28), a random effects model was applied to determine the pooled prevalence of family planning utilization. Meta-regression and subgroup analysis were also used to identify the source of heterogeneity across studies by using year of study, and region. To check for publication bias, a funnel plot and Egger's test were used (29). The statistical significance of publication bias was determined using a p-value less than 0.05 (30). Texts, tables, and forest plots with effect and 95% CI measures were used to present the results.

3. **Result**
   3.1. **Study selection**

Using various search strategies, a total of 2080 studies were retrieved from various international databases and Ethiopian university institutional repositories. All retrieved studies were screened using the Endnote 7 reference manager, and 1421 were removed due to duplication. Then, 652 studies were eliminated due to unrelated titles, abstracts, inaccessibility of full text, and differences in outcomes of interest. Finally, for the meta-analysis, seven studies that met the inclusion criteria were considered (**Figure 1**).

3.2. **Characteristics of included studies**

This systematic review and meta-analysis included seven primary studies. All of the studies included were cross-sectional, with sample sizes ranging from 162-701(25,31). Regarding the study's region, three articles were from the Amhara region (23,25,32), two from Addis Ababa (31,33), one from SNNP (34) and one from the Tigray (24) region with years of study ranging from 2013 to 2020. The highest prevalence of family planning utilization was reported by Abera S. (44.4%)(31), which was done in the Addis Ababa, while the lowest utilization was reported in the Amhara region by Beyene GA, et al. (15.8%) (23) (**Table 1**).

Table 1: Characteristics of the included studies in the systematic review and meta-analysis

| Authors Name | Publication Year | Study setting | Region | Study design | sample | prevalence%  (95% CI) |
| --- | --- | --- | --- | --- | --- | --- |
| Yesgat YM, et al. | 2019 | Arba Minch | SNNPR | Cross Sectional | 398 | 33.7 (29.06, 38.34) |
| Mekonnen AG, et al. | 2019 | Debre Berhan | Amhara | Cross Sectional | 397 | 24.5 (20.27, 28.73) |
| Beyene GA, et al. | 2013 | Gondar | Amhara | Cross Sectional | 267 | 15.8 (11.42, 20.18) |
| Yimer AS, et al. | 2017 | Addis Ababa | Addis Ababa | Cross Sectional | 326 | 31.1 (26.07, 36.13) |
| Abera S (upulished) | 2016 | Addis Ababa | Addis Ababa | Cross Sectional | 701 | 44.4 (40.72, 48.08) |
| Tilahun A (upulished) | 2020 | Bahir Dar | Amhara | Cross Sectional | 162 | 30.2 (23.13, 37.27) |
| Tsegay K, et al. | 2013 | Mekelle | Tigray | Cross Sectional | 536 | 27.2 (23.43, 30.97) |

3.3. **Prevalence of family planning utilization among women with disability**

A total of 7 (five published and two unpublished) studies with 2787 women were included in this systematic review and meta-analysis to estimate the pooled prevalence of family planning utilization among women with disabilities in Ethiopia. Accordingly, the overall estimated pooled prevalence of family planning utilization among women with disabilities with a random effects model was 29.57% (95% CI: 22.30, 36.83) with a heterogeneity index (I^2^) of 94.6% (p = 0.000) **(Figure 2).**

3.4. **Publication bias and Heterogeneity**

To check for publication bias, a funnel plot and Egger's test (28) were used. The statistical significance of publication bias was determined using a p-value less than 0.05(29). Accordingly, the funnel plot results revealed a symmetrical pattern, indicating that the included studies do not have a publishing bias **(Figure 3).** Furthermore, Egger's regression test is not significant, indicating that there was no publication bias in the studies **(Table 2).**

Table 2: Egger's regression test to show publication bias in the studies

| Std.Eff | Coef. | Std. Err. | T | p> t | [95% Conf. Interval] |
| --- | --- | --- | --- | --- | --- |
| Slope | 3.587349 | 0.7756355 | 4.63 | 0.006 | 1.593514,5.581183 |
| Bias | -0.1038034 | 0.3440247 | -0.30 | 0.775 | -0.988147,0.7805403 |

3.5. **Subgroup analysis and Sensitivity analysis**

Significant heterogeneity was observed among included primary studies. To identify the source of heterogeneity, sub-group analysis was performed based on the region and year of study. As a result, the overall prevalence of family planning utilization was found to be high in Addis Ababa region studies [32.61, (26.39, 38.82)] **(Figure 4A)** and studies conducted after 2015 [32.61, (26.39, 38.82)] **(Figure 4B).**

A leave-one-out sensitivity analysis was done to check the effect of individual studies on the pooled estimate of family planning utilization. The result indicated removing a single study did not have a significant influence on pooled prevalence and the pooled prevalence of family planning utilization was observed low at 25.63% (3.93, 167.08%) and high at 31.78% (5.22, 193.66%) when Abera S. and Beyene GA, et al. were omitted respectively **(Table 3).**

Table 3: Sensitivity analysis for Utilization of Family Planning among Women with Disability in Ethiopia

| Study omitted | Year of study | Pooled estimate (%) | 95%CI |
| --- | --- | --- | --- |
| Yesgat YM, et al. | 2019 | 28.03 | 4.68,167.89 |
| Mekonnen AG, et al. | 2019 | 29.56 | 4.8, 182.05 |
| Beyene GA, et al. | 2013 | 31.79 | 5.22, 193.66 |
| Yimer AS, et al. | 2017 | 28.42 | 4.82, 167.02 |
| Abera S (upulished) | 2016 | 25.63 | 3.93, 167.08 |
| Tilahun A (upulished) | 2020 | 28.62 | 5.13, 159.65 |
| Tsegay K, et al. | 2013 | 29.09 | 4.51 , 187.48 |

3.6. **Factors associated with family planning utilization among women with disability**

Three variables (Age, Marital status, and Discussion with partner) were extracted to identify factors associated with family planning utilization among women with disability. Of this marital status and discussion with a partner were found to be significantly associated with family planning utilization among WWDs **(Table 4).** However, there was no statistically significant association between women's age and FP utilization**.**

The pooled effect of the marital status on FP utilization among women with disabilities was evaluated by using three primary studies (24,33,34) The result of this study revealed that marital status was significantly associated with FP utilization and the likelihood of utilizing FP was 8.6 times higher among those women who had in marital union than their counterparts [OR: 8.63; 95% CI (3.17, 23.46); P<0.001], with heterogeneity (I^2^ = 91.5%, p-value <0.001) **(Figure 5).**

To determine the pooled effects of women's discussions with their partners on FP utilization, two studies were included (25,31) The findings revealed that women's discussion with their partners was significantly associated with the use of FP; those who discussed with their partner were 6 times more likely to use FP than those who did not [OR: 6.08; 95% CI (1.64, 22.53; P=0.007)] with heterogeneity (I^2^ = 84.2%, p-value = 0.012) **(Figure 6).**

Table 4: Factors associated with Utilization of Family Planning among Women with Disability in Ethiopia

| Determinants | Comparisons | | Number of studies | Sample size | OR(95% CI) | P- value | I^2^ (%) | Heterogeneity test(P- value) |
| --- | --- | --- | --- | --- | --- | --- | --- | --- |
| Marital Status | | married Vs. unmarried | 3 | 1260 | 8.63(3.17‒23.46) | <0.001* | 91.5 | <0.001 |
| Age | | <25 Vs. ≥ 25 | 2 | 593 | 2.89(0.95‒8.81) | 0.062 | 81.3 | 0.021 |
| Discussion with partner | | Yes Vs. No | 2 | 354 | 6.08(1.64‒22.53) | 0.007* | 84.2 | 0.012 |

*Significant level <0.05

4. **Discussion**

The purpose of this systematic review and meta-analysis was to determine the pooled prevalence and associated factors of FP utilization among Ethiopian women with disabilities. To the best of our knowledge, this meta-analysis is the first of its kind in determining the national prevalence and significant factors of FP utilization among WWDs in Ethiopia, which will be used as input for policymakers, health care providers, and other stakeholders in developing evidence-based strategies to improve FP utilization among WWDs.

The overall pooled prevalence of family planning utilization among women with disabilities in Ethiopia was 29.57% (95% CI: 22.30, 36.83), which is lower than the Ethiopian Demographic and Health Survey (EDHS) report 2019 (35). This could be because the EDHS reports family planning utilization among all women, but this review was limited to WWDs. Also, it is lower than a review conducted in Ethiopia (36,37). The difference might be due to the difference in the study period of primary studies and the study population, since the two studies are conducted among adolescents and postpartum women who are more information about sexual and reproductive health from different sources.

The result of this review was higher than the minimum rate of family planning utilization among WWDs, according to a review conducted by Horner-Johnson (38) and Beyene GA (18). The result of this study could be a polled estimate of different studies, whereas Horner-Johnson and Beyene GA report the minimum and maximum range of family planning utilisation among WWDs by reviewing literature from different settings, and the comparison in this study was based on the minimum rage from the report.

Based on the subgroup analysis result, the prevalence of family planning utilization was found to be high in Addis Ababa region studies [32.61, (26.39, 38.82)]. This could be attributed to the city's urbanization and women's increased awareness. Because Addis Ababa is the country's capital, disabled women may have more access to information on family planning. . In Addis Ababa, there is also the Ethiopian Women with Disability Association, where many women participate and discuss their health, including family planning.

In addition, this review revealed that there is a high prevalence of family planning utilization among studies conducted after 2015 [32.61, (26.39, 38.82)]**.** The reason could be that after the Millennium Development Goals (MDG) were completed in 2015; there was an increase in awareness about maternal and reproductive health services usage including family planning. Furthermore, various information distribution technologies such as social media are increasingly being used to disseminate information regarding family planning options. This may make it easier for women to learn about the advantages of family planning, where to get it, and how to use it.

In this review, the odds of utilizing FP were 8.6 times higher among women who had been in a marital union than their counterparts. This is in line with a review conducted in sub-Saharan Africa (39), Ethiopia (40) and a report from USAID(41) . This is because family planning can increase partners' involvement in decisions about whether and when to have children while also assisting them in avoiding unintended pregnancy. It also provides her with enough time and opportunity to love and care for her husband and children. As a result, partner involvement in family planning decisions is critical, and a married woman may be encouraged to use contraception by her partner.

The findings of this study also revealed that women who discussed with their partner were 6 times more likely to use FP than those who did not. This is consistent with the review conducted in Sub-Saharan Africa (39,42). This may be due to the fact that couples who talk about FP issues are more likely to jointly decide on the type of contraceptive method to use, the number of children to have, and the spacing between the children. As a result, they are more likely to use the service. In addition male involvement plays a role in the use of reproductive and maternal health services, and any factor that influences the partner's attitude towards these services will have an effect on women's use, either positively or negatively. So talking with a partner may give them the impression that she values their influence in her life, giving the woman more freedom to make decisions.

There are limitations to this study. The absence of studies from some Ethiopian regions in this study makes it challenging to extrapolate the results to the national level. Second, the results should be interpreted cautiously due to the significant heterogeneity among the studies. Finally, we found it challenging to compare our results because there were few systematic reviews and meta-analyses conducted at the national, regional, and international levels.

5. **Conclusion**

According to the findings of this study, only one-third of disabled women in Ethiopia utilize family planning. Being married and discussing with a partner were significantly associated with family planning utilization among WWDs. It is important to increase access to quality contraceptive care and improve negative clinician attitudes and awareness of disabled women to increase utilization. In addition, it is critical to pay more attention to the reproductive health care needs of women with disabilities to improve health care equity and quality.

**Abbreviations**

| CI | Confidence Interval |
| --- | --- |
| EDHS | Ethiopian Demographic and Health Survey |
| FP | Family Planning |
| MDG | Millennium Development Goals |
| SNNPR | Southern Nations, Nationalities and Peoples Region |
| WHO | World Health Organization |
| WWDs | Women with Disabilities |
| USAID | U.S. Agency for International Development |

**Authors’ contributions**

TL: conceived and designed the review, and was the guarantor of the review. MA, TM, and MSC developed the search strings, and screened, selected, and evaluated the quality of the studies. TL, MS, and BBT extracted the data from selected studies, carried out analysis, and interpretation, and rigorously reviewed the manuscript. TL carried out the draft of the manuscript. All authors read and approved the final version of the manuscript.

**Ethical approval and consent to participate**

Not applicable. This study estimated the pooled prevalence of family planning utilization from the already published primary studies and grey literature. So, we did not use primary data that needs ethical approval and consent to participate. However, ethical issues such as plagiarism, misconduct, data fabrication/falsification, double submission/publication, and so on have been seriously observed by the authors during the data extraction, manuscript writing, and submission.

**Consent for publication:** Not applicable.

**Competing interests:** The author(s) declared no potential conflicts of interest with respect to the research, authorship, and/or publication of this article.

**Funding:** The author(s) received no financial support for the research, authorship, and/or publication of this article.

**Availability of data and materials**

All the datasets used in this study are accessible in the published article and its Additional files.

6. **Reference**

1.	Disabled World. Disabilities: Definition, Types and Models of Disability - Disabled World [Internet]. 2019. p. 1–3. Available from: https://www.disabled-world.com/disability/types/

2.	World Health Organization (WHO). Disability and health [Internet]. 2021. Available from: https://www.who.int/news-room/fact-sheets/detail/disability-and-health

3.	UN WOMEN. MAKING THE SDGS COUNT FOR WOMEN AND GIRLS WITH. 2017.

4.	Qaisar M. History of Family Planning and Role of ICFP 2022 _ ICFP. In: interational confrence on family planning. 2022.

5.	UNFPA. Family planning. Vol. 55, Indian medical journal. 2021. p. 259–60.

6.	World Health Organization (WHO). Family Planning / Contraception Methods Key facts [Internet]. Who.Int. 2020. p. 1–5. Available from: https://www.who.int/news-room/fact-sheets/detail/family-planning-contraception

7.	United Nations. World Contraception Day 2022: Access to safe and effective family planning methods key to gender equality and improved livelihoods _ United Nations in Timor-Leste. 2022.

8.	UNFPA. Women and young people with disabilities_ A needs assessment of sexual and reproductive health and rights, gender-based violence, and access to essential services - Samoa - Samoa _ Relie. 2022.

9.	World Health Organization, Johns Hopkins USA for ID. Family Planning A GLOBAL HANDBOOK FOR PROVIDERS? 2018. 327–328 p.

10.	WHO, UNFPA. Promoting sexual and reproductive health for persons with disabilities. 2009.

11.	Ledingham E, Wright GW, Mitra M. Sexual Violence Against Women With Disabilities: Experiences With Force and Lifetime Risk. Am J Prev Med [Internet]. 2022;62(6):895–902. Available from: https://doi.org/10.1016/j.amepre.2021.12.015

12.	Beaudrap P De, Mouté C, Pasquier E, Tchoumkeu A, Temgoua CD, Zerbo A, et al. Burden of and risk factors for sexual violence among women with and without disabilities in two sub-Saharan African countries. Glob Health Action [Internet]. 2022;15(1). Available from: https://doi.org/10.1080/16549716.2022.2077904

13.	Report W. Fact Sheet : Violence against Women and Girls with Disabilities. 2013;(February).

14.	Horner-johnson W, Klein KA, Campbell J, Guise J. Experiences of Women With Disabilities in Accessing and Receiving Contraceptive Care. J Obstet Gynecol Neonatal Nurs [Internet]. 2021;50(6):732–41. Available from: https://doi.org/10.1016/j.jogn.2021.07.005

15.	MacQuarrie KLD, Julia Fleuret. Patterns of Reproductive Health among Women with Disabilities. In: DHS ANALYTICAL STUDIES. 2022. p. 80.

16.	Tesfaye T, Markos EW, Chea N, Wachamo D. Accessing Healthcare Services for People with Physical Disabilities in Hawassa City Administration , Ethiopia : A Cross-Sectional Study. Risk Manag Healthc Policy. 2021;14:3993–4002.

17.	Soule O, Sonko D. Examining access to sexual and reproductive health services and information for young women with disabilities in Senegal : a qualitative study Examining access to sexual and reproductive health services and qualitative study. 2022;

18.	Beyene GA. Contraceptive Dynamics Among Women with Disabilities in Low-and Middle-Income Countries : A Scoping Review Protocol Registration : 2021;1–12.

19.	Moher D, Liberati A, Tetzlaff J, Altman DG. Preferred reporting items for systematic reviews and meta-analyses: The PRISMA statement. BMJ [Internet]. 2009;339(7716):332–6. Available from: http://dx.doi.org/doi:10.1136/bmj.b2535

20.	Liberati A, Altman DG, Tetzlaff J, Mulrow C, Gøtzsche PC, Ioannidis JPA, et al. The PRISMA statement for reporting systematic reviews and meta-analyses of studies that evaluate health care interventions: explanation and elaboration. Vol. 62, Journal of clinical epidemiology. 2009. 1–34 p.

21.	Modesti PA, Reboldi G, Cappuccio FP, Agyemang C, Remuzzi G, Rapi S, et al. Cross Sectional Study Newcastle - Ottawa Quality Assessment Scale. PLoS One [Internet]. 2016;11(1):1–2. Available from: https://journals.plos.org/plosone/article/file?type=supplementary&id=info:doi/10.1371/journal.pone.0147601.s001

22.	Kassa BG, Ayele AD, Belay HG, Tefera AG, Tiruneh GA, Ayenew NT, et al. Postpartum intrauterine contraceptive device use and its associated factors in Ethiopia: systematic review and meta-analysis. Reprod Health. 2021;18(1):1–12.

23.	Beyene GA, Munea AM, Fekadu GA. Modern contraceptive use and associated factors among women with disabilities in gondar city, amhara region, north west ethiopia: A cross sectional study. Afr J Reprod Health. 2019;23(2):101–9.

24.	Kellali T, Hadush G FH. Modern Contraceptive Methods Utilization and Associated Factors among Women with Disabilities. Int J Pharm Biol Sci Fundam [Internet]. 2017;13(01)(01):1–8. Available from: www.ijpbsf.com

25.	Amsaya T. Modern Contraceptive Utilization and Associated Factors Among Disabled and Non Disabled Reproductive Age Group Women in Bahir Dar City , Ethiopia. 2021;

26.	Higgins JPT, Thompson SG, Deeks JJ, Altman DG. Measuring inconsistency in meta-analyses. Br Med J. 2003;327(7414):557–60.

27.	Borenstein M, Hedges L V., Higgins JPT, Rothstein HR. A basic introduction to fixed-effect and random-effects models for meta-analysis. Res Synth Methods. 2010;1(2):97–111.

28.	DerSimonian R, Kacker R. Random-effects model for meta-analysis of clinical trials: An update. Contemp Clin Trials. 2007 Feb;28(2):105–14.

29.	Rothstein HR, Sutton AJ, Borenstein M. Comparison of Two Methods to Detect Publication Bias in Meta-analysis. Am Med Assoc. 2006;295(6):1–7.

30.	Begg CB, Mazumdar M. Operating Characteristics of a Rank Correlation Test for Publication Bias. Biometrics. 1994;50(4):1088.

31.	Abera S. The Assessment of Determinants of family planning use and unmet need among women of reproductive age group with disabilities in Addis Ababa. 2016;(November).

32.	Mekonnen AG, Bayleyegn AD, Aynalem YA, Adane TD, Muluneh MA, Asefa M. Level of knowledge, attitude, and practice of family planning and associated factors among disabled persons, north-shewa zone, Amhara regional state, Ethiopia. Contracept Reprod Med. 2020;5(1):1–7.

33.	Yimer AS, Modiba LM. Modern contraceptive methods knowledge and practice among blind and deaf women in Ethiopia. A cross-sectional survey. BMC Womens Health. 2019;19(1):1–13.

34.	Mesfin Yesgat Y, Gebremeskel F, Estifanous W, Gizachew Y, Jemal S, Atnafu N, et al. <p>Utilization of Family Planning Methods and Associated Factors Among Reproductive-Age Women with Disability in Arba Minch Town, Southern Ethiopia</p>. Open Access J Contracept. 2020;Volume 11:25–32.

35.	Ethiopian Public Health Institute (EPHI) [Ethiopia], ICF. Ethiopia Mini Demographic and Health Survey 2019: Key indicators. 2019.

36.	Wake GE, Fitie GW, Tizazu MA. A Systematic Review and Meta-Analysis on Post-Abortion Contraceptive Utilization and Associated Factors in Ethiopia. Front Public Heal. 2022;10(May).

37.	Mekonnen AG, Odo DB, Nigatu D. Review Article Adolescents ’ Contraceptive Uptake in Ethiopia : A Meta-Analysis. 2022;2022.

38.	Horner-Johnson W, Moe EL, Stoner RC, Klein KA, Edelman AB, Eden KB, et al. Contraceptive knowledge and use among women with intellectual, physical, or sensory disabilities: A systematic review. Disabil Health J [Internet]. 2019;12(2):139–54. Available from: https://doi.org/10.1016/j.dhjo.2018.11.006

39.	Blackstone SR, Nwaozuru U, Iwelunmor J. Factors Influencing Contraceptive Use in Sub-Saharan Africa : A Systematic Review. 2017;

40.	Jima GH, Kaso MK, Biesma-Blanco RG, Sendekie TY, Stekelenburg J. Factors associated with modern contraceptives uptake during the first year after birth in Ethiopia: A systematic review and meta-analysis. PLoS One. 2023;18(2 February).

41.	USAID. CONTRACEPTIVE USE , AND UNMET DHS COMPARATIVE. 2017;(July).

42.	Souza PD, Bailey J V, Stephenson J, Oliver S, Souza PD, Bailey J V, et al. Factors influencing contraception choice and use globally : a synthesis of systematic reviews. Eur J Contracept Reprod Heal Care [Internet]. 2022;27(5):364–72. Available from: https://doi.org/10.1080/13625187.2022.2096215

**Figure Legends**

Figure 1: PRISMA flow diagram of study selection

Figure 2. Prevalence of family planning utilization among women with disability in Ethiopia

Figure 3: Funnel plot to test the publication bias in 7 studies with 95% confidence limits

Figure 4A. Subgroup analysis based on the region among women with disability in Ethiopia

Figure 4B. Subgroup analysis based on year of study among women with disability in

Ethiopia

Figure 5: The pooled effect of marital union on FP utilization among women with disabilities

in Ethiopia.

Figure 6: The pooled effect of women's discussions with their partners on FP utilization

among women with disabilities in Ethiopia.
