# Supplementary material for: Utilization of family planning and associated factors among women with disabilities in ethiopia: A systematic review and meta-analysis
Source: PLoS One. 2023 Sep 8;18(9):e0291189. doi: 10.1371/journal.pone.0291189 (PMC10490906; doi:10.1371/journal.pone.0291189)
Supplement: Attachment — Submitted filename: Second Revision Response to the Editor.docx [file pone.0291189.s006.docx]

The authors would like to thank the editorial team for constructive and valuable comments. The authors are very happy to submit the revised version of the manuscript entitled “Utilization of Family Planning and Associated Factors among Women with Disabilities in Ethiopia: a systematic review and meta-analysis” for its publication in your Journal. The comments were highly insightful and enabled us to greatly improve the quality of our manuscript. In this revised manuscript we made substantial changes to address your concerns in a point-by-point response. We are very keen to incorporate further comments, if any, for the betterment of the final manuscript.

1. **Comment [A1]:** As I have said in the previous comment the identified articles from each data base is needed…..PubMed=-----?, CINARI=----? For each databases

**Answer –**Thank you for your comment; it is accepted and corrected in the revised manuscript.

2. **Comment [A2]:** I haven’t seen the logical evidences to exclude case control study in this review.

**Answer –** Thank you for your inquiry; First the authors plan to include all observational studies but all studies who fulfil the eligibility criteria in this review were crossectional, that’s why the authors don’t included other observational studies like case control studies

3. **Comment [A3]:** What is your standard to say this finding is low?

**Answer –** According to the Ethiopian Demographic Health Survey 2019, this finding is low.

4. **Comment [A4]:** Have you got in your finding which showed health care equity and quality were the main determinant factors for low FP service utilization?

**Answer –** Thank you for your comment; it is accepted and corrected in the revised manuscript.

5. **Comment [A5 & A6]:** This study aimed to determine the pooled prevalence and associated factors for FP service utilization among WWDs in Ethiopia; better to state it at the end of the last paragraph of the introduction.

**Answer –** Thank you for your comment; it is accepted and corrected in the revised manuscript.

6. **Comment [A7]:** Thank you for your comment; it is accepted and corrected in the revised manuscript.
7. **Comment [A8]:** Which articles were included or excluded based on this reason.

**Answer –** Thank you for your inquiry; the included and excluded studies are cited in the revised manuscript.

8. **Comment [A9]:** Thank you for your comment; it is accepted and corrected in the revised manuscript.
9. **Comment [A10]:** Is there any logical evidence to exclude a single variable which showed significant association in the single study?

**Answer–** Even though there is no clear evidence to exclude a single variable that showed a significant association in the single study, meta-regression is the aggregate result of two or more studies. So to get the pooled results of different studies, the authors preferred to extract factors that were reported in two or more studies.

10. **Comment [A11]:** Better to state all variables even they don’t have significant association

**Answer–** Thank you for your comment; as we mentioned earlier in this study the variable was extracted as factors, if it is significant associations in two or more primary papers. So, in this study only three variables were extracted as a factor and among them two of them are significantly associated with the dependent variable which is seen in **Table 4.**

11. **Comment [A13]:** You have used very limited literatures for discussion.

**Answer –** Thank you for your comment; it is accepted and corrected in the revised manuscript.

12. **Comment [A14]:** Thank you for your comment; it is accepted and corrected in the revised manuscript.
