# Supplementary material for: Utilization of family planning and associated factors among women with disabilities in ethiopia: A systematic review and meta-analysis
Source: PLoS One. 2023 Sep 8;18(9):e0291189. doi: 10.1371/journal.pone.0291189 (PMC10490906; doi:10.1371/journal.pone.0291189)
Supplement: Attachment — Submitted filename: Response to the Editor 3.docx [file pone.0291189.s007.docx]

**Response to the Editor**

1. The reason to exclude case control studies not convincing.

**Answer:** Thank you for your comment, as the authors mentioned in the manuscript on page 5 lines 2-4, both published and unpublished observational studies in English that report the prevalence and/or associated factors of family planning utilization among disabled women in Ethiopia were considered. But all studies which fulfill the inclusion criteria are crossectional, that’s why all the included studies were crossectional.

2. Excluding variables which are significant in the single study is not advisable; we can take odds ration even though it may be not significant.

**Answer:** Thank you for your comment, its accepted and corrected as your kind recommendation in the revised manuscript on pages 9 and 10.
